# Are Allergy-Induced Implant Failures Actually Hypersensitivity Reactions to Titanium? A Literature Review

**DOI:** 10.3390/dj11110263

**Published:** 2023-11-09

**Authors:** Megumi Watanabe, Lipei Liu, Tetsuo Ichikawa

**Affiliations:** Department of Prosthodontics & Oral Rehabilitation, Tokushima University, Graduate School of Biomedical Sciences, 3-18-15, Kuramoto, Tokushima 770-8504, Japan; agnes_dango@foxmail.com (L.L.); ichi@tokushima-u.ac.jp (T.I.)

**Keywords:** titanium allergy, implant failure, clinical examination

## Abstract

Purpose: This literature review was performed to assess whether implant failures are associated with titanium allergy. Materials and Methods: An electronic search of the MEDLINE/PubMed, Cochrane Library, and Scopus databases up to April 2021 was conducted, and the obtained articles were independently assessed by two reviewers. Articles describing cases of implant failure in which the cause of implant failure was only identified as allergy were included. Results: Twelve studies were included. Eight studies identified Ti allergy by clinical examinations, of which four used patch tests, three used the lymphocyte transformation test (LTT)/memory lymphocyte immunostimulation assay (MELISA), and one used both tests. Nine studies reported cases of titanium hypersensitivity in combination with other systemic allergy-related disorders, with eight cases also showing positive results for Ni, Hg, Cr, and Co hypersensitivity. Ten papers reported the improvement of symptoms after the removal of the Ti implants and their replacement with zirconia implants, and two of these papers showed good results. Conclusion: Cases of probable titanium allergy included those with true titanium allergies and those with a potentially different cause. However, the differentiation of these cases is difficult. Since no definitive method has been established for diagnosing titanium allergy, a comprehensive diagnosis based on the clinical course and clinical examination using a patch test/LTT/MELISA is necessary. Implant treatment should be performed with caution in patients with any preoperative allergies.

## 1. Introduction

Dental metal allergy is one of the problems caused by the use of metals in dentistry. While mercury and nickel are well-known antigenic metals that can trigger allergic reactions, hypersensitivity reactions to titanium have recently been reported, especially in the field of orthopedics [1,2,3,4]. Because titanium shows excellent biocompatibility and is thought to not cause immune reactions in the host, titanium dental implants have been used as a safe and predictable treatment option for more than 50 years [5]. Their safety can be attributed to the high biocompatibility of titanium, since titanium implants continue to exist safely in the patient’s bone in harmony with a healthy organism and excellent biomaterials [6].

Although unlike dynamically moving joints, implants placed in the jawbone are considered to be in a relatively immobile state, so that the risk of titanium powder adhering to bone or soft tissue over time is considered to be lower compared to artificial joints, hypersensitivity reactions potentially caused by dental implants have also been reported. The causes of implant failure include bone tissue damage due to poor surgical techniques, bacterial infections, host factors such as poor healing and poor bone quality, poor prosthesis design, and excessive or traumatic occlusal loading on the implants [7], and these failures cannot be attributed to the use of titanium itself. However, unexplained implant failures may occur despite the absence of these issues, and rejection reactions or allergic responses to the implant may be a potential cause.

In the present study, based on the possibility that dental implants, which are a commonly employed treatment using titanium in dentistry, may cause hypersensitivity reactions, we reviewed case-reports to determine whether implant failures could be attributed to titanium hypersensitivity. Furthermore, we examined the scientific validity of titanium hypersensitivity diagnosis.

## 2. Materials and Methods

The focused question to conduct a literature search was as follows: Does titanium hypersensitivity cause dental implant treatment failure?

### 2.1. Search Strategy

Various combinations of the following terms were utilized for the data search: “dental implants” (MeSH Terms), “hypersensitivity” (MeSH Terms), “allergy” (All Fields), “implant lost” AND “implant failure” (All Fields). The search was performed in the MEDLINE/ PubMed, Cochrane Library, and Scopus databases and limited to articles written in English and published until 2021.

### 2.2. Inclusion/Exclusion Criteria

Articles that contained information on implant defects or bone resorption around implants and presented cases in which the cause of implant failure was only reported as allergy, regardless of the type of allergy, were selected. Review articles, articles that were not written in English, and articles that described obvious causes of implant failure, such as infection or mechanical stress, dealt with non-dental implants, or included only basic research without cases were excluded.

### 2.3. Data Collection

A literature search was performed, and its findings were independently evaluated by two authors (M.W. and L.L.). The two reviewers selected abstracts based on the criteria listed above, and the full text of papers that met these criteria was read to determine if the papers reported detailed information.

## 3. Results

### 3.1. Characteristics of the Representative Articles

**Figure 1:**
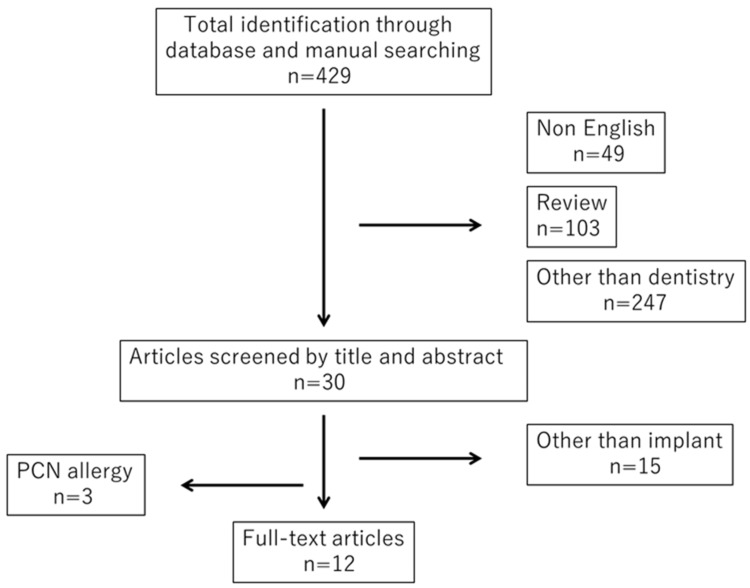
PRISMA diagram.

Thirty articles were screened for their potential relevance by manual searching, and their contents were examined in detail. Twelve articles were selected on the basis of the selection protocol (Figure 1). The selected articles are summarized in Table 1 and Table 2 [8,9,10,11,12,13,14,15,16,17,18,19].

Eight articles revealed an allergy to titanium on clinical examination. Of these, four described diagnosis using a patch test, three used the lymphocyte transformation test (LTT)/memory lymphocyte immunostimulation assay (MELISA), and one used both tests. Allergy was determined by immunohistochemistry (IHC) in two cases and by clinical symptoms alone in two cases. The most common symptoms of titanium allergies were eczema and gingival hyperplasia. Other symptoms included cheilitis, swelling, and mucosal pain. Table 2 summarizes nine case-reports. Symptoms arose weeks to months after the implant placement. Erythema and swelling of the face skin and gingiva were observed immediately after implant placement in some cases.

### 3.2. Positive Results for Allergies Other Than Titanium

Among the articles selected for this review, nine reported cases of hypersensitivity to titanium that also involved other generalized allergy-related disorders, and the patients in eight articles were also positive for metal allergies other than titanium. Although one patient was allergic to pierced earrings, and the causative metal was not clear, the other seven patients showed hypersensitivity reactions to nickel (Ni), mercury (Hg), chromium (Cr), and cobalt (Co). And also tested positive to these metals in many cases (Table 3).

### 3.3. Articles on Penicillin Allergy

In ten articles, the removal of Ti implants improved allergic symptoms, and in two of these articles, the replacement of Ti implants with zirconia implants showed good results [17,18]. Three articles that were excluded after reading the abstract described the risk of potential penicillin allergy in implant therapy (Table 4) [20,21,22].

## 4. Discussion

Firstly, we evaluated the possibility that allergies, especially titanium allergy, can cause implant failure on the basis of case-reports on actual hypersensitivity reactions. In cases of implant failure without obvious causes such as infection or overload, host rejection may be the reason for implant failure. The patients in the case-reports and retrospective studies selected in this review may be examples of host rejection. However, as stated in this section, the lack of a clear mechanism for intraoral eluted titanium and a validated test method for titanium allergy are limitations to further research.

### 4.1. Mechanism of Dental Metal Allergy Development

Metal allergy is a delayed type of hypersensitive reaction and is classified as type IV in the allergy classification proposed by Coombs and Gell (Table 5) [23]. Delayed-type hypersensitivity reactions are those in which symptoms are observed within 24 to 48 h after contact with the antigen, due to the involvement of cellular immune responses by T lymphocytes.

In many cases, dental metal allergy occurs when metal restorations are placed in the oral cavity after metal in jewelry or cosmetics has been taken in through the skin and the patient becomes sensitized. Titanium is a material with high biocompatibility, has been used for dental implants for more than half a century [24], and is widely known to have shown very good clinical results. However, in recent years, there have been reports of cases of allergy to titanium. This may be due to the increased exposure to titanium. Titanium is included in cosmetics such as sunscreen and foundation, and is also used in pierced earrings as a safe alternative to nickel and other metals [25,26,27]. In other words, the opportunities to come into contact with titanium have been increasing in recent years, and it is not surprising that the body has become sensitized to titanium as it has been to other metals that have been allergens in the past [28].

**Figure 2:**
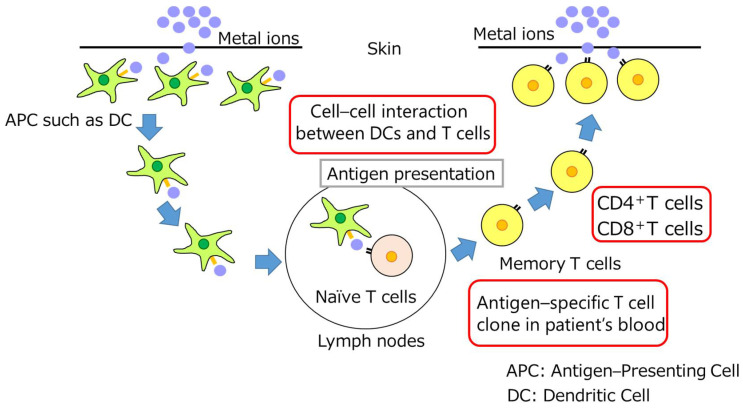
Possible mechanism of sensitization and elicitation. T cells: T cells are a type of lymphocyte that is involved in cellular immunity related to metal allergy. APC:-Antigen presenting cells. DC: Dendritic cells are the most professional potent antigen-presenting cells.

The pathogenesis of metal allergy is established through a sensitization phase and an elicitation phase (Figure 2). The allergic reaction begins when a small hapten of 1 kDa or less adheres to the skin or mucous membranes, and it is not clear whether this hapten is a metal ion, a protein denatured by the metal, or something else. Clinically, however, it is thought that the dissolution (ionization) of metals by perspiration is important for the process of skin passage [29].

Several hours after adhering to the skin or mucous membranes, haptens pass through the keratinocyte layer of the epidermis and infiltrate into the subepithelium [30,31]. At this time, keratinocytes produce IL-1β, TNF-α, prostaglandin E2, etc., in response to haptens and activate antigen-presenting cells such as dendritic cells. Antigen-presenting cells that capture haptens migrate to local lymph nodes and present antigens to T cells via the major histocompatibility complex; the MHC and T-cell receptors thereby induce antigen-specific CD4-positive T cells and CD8-positive T cells. T cells that have memorized the antigen become memory T cells and prepare for the next antigen invasion. This is called sensitization, which usually takes one to two weeks. When the same hapten invades a sensitized individual again, keratinocytes produce cytokines that activate antigen-presenting cells, which in turn present antigens to T cells. In addition, existing memory T cells, i.e., antigen-specific T cells, respond rapidly to the hapten, triggering an inflammatory response. The elicitation phase lasts from 48 to 72 h. Thus, the interaction between keratinocytes, antigen-presenting cells, and T cells plays an important role in the development of metal allergy [32].

One of the factors that contribute to titanium’s excellent biocompatibility is that it is a very stable metal. Titanium is thought to have a passive oxide film on its surface that makes it difficult to ionize and pass through the skin [33]; however, the dynamics of titanium when skin is in direct contact with the titanium surface for a long period of time, such as with pierced skin, is unknown. It is not clear whether titanium is really stable in the harsh environment of the oral cavity. In the oral environment, titanium is constantly exposed to liquid components and proteins in saliva and food under body temperature; it is also exposed to H2S, an acid favored by bacteria; it is easily ionized by galvanic current generation due to coexistence with other types of metals; it is always in a crevice corrosion environment in the narrow gap between adjacent teeth; and it is subject to metal. The chloride ions in the tissue fluid exuding from the gingival sulcus and gingival pockets and in foodstuffs may destroy the passive oxide film. In addition, titanium particles are also taken into the body at the time of implantation, since dental implants are placed into bone through the mucosa. In other words, the increase in clinical reports of titanium allergy cases in recent years can be attributed to the increasing opportunities to become sensitized to titanium in daily life and the widespread use of titanium in dental treatment, as represented by dental implants, regardless of whether the reaction is truly allergic or not.

### 4.2. Clinical Symptoms of Titanium Allergy

Erythema, dermatitis, and local swelling were identified as the clinical manifestations of probable titanium allergy in this review. Itching, burning, and pain have also been reported in some cases. Nevertheless, since hypersensitivity reactions to titanium have been reported in patients with postoperative complaints [34], a more reliable method for the diagnosis of titanium allergy is required to rule out other non-allergic factors as the causes of clinical symptoms. Although titanium must be proven to be the allergen to exclude other non-allergic factors as the cause of clinical symptoms, there is no established method to reliably diagnose titanium allergy. Currently, the diagnosis of suspected hypersensitivity reactions to titanium is based on an assessment of the clinical course, symptoms, application of conventional metal allergy tests, and the effect of treatments such as implant removal.

### 4.3. Immunological Mechanisms of Metal-Allergy-Testing Methodologies 

**Figure 3:**
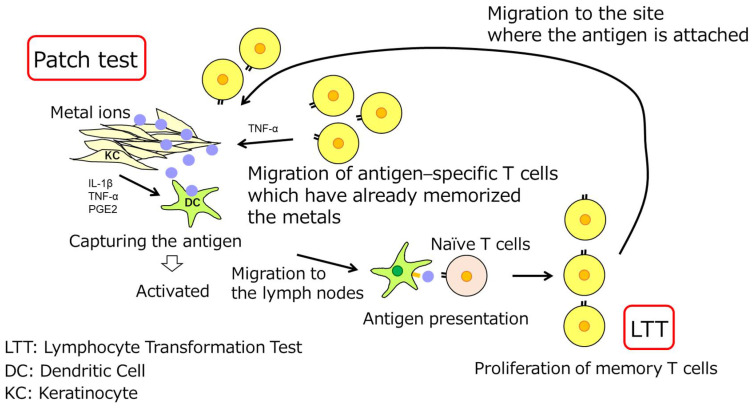
Immunological mechanisms of patch test and LTT.

Metal allergy is a cellular immune reaction centered on T cells, and an immune response is triggered when metal sensitized on the skin is contained in a restoration or cement placed in the oral cavity. The patch test reproduces this in vivo (Figure 3). When a metal reagent is applied to a patient’s back skin, memory T cells, that have already been sensitized to the metal and have stored the antigen in the patient’s body, gather around the metal and produce inflammation such as redness and swelling by cytokine production on the skin, which is judged as positive. On the other hand, if there are no memory T cells for the metal in the body, no inflammation occurs, and the test is negative. Although the patch test is the most reliable examination method currently available, false-positive and false-negative results are possible, and the interpretation of the results is often difficult, so thorough knowledge is required. There is also a risk of new sensitization by the application of metallic reagents. Furthermore, there is a risk of temporary allergic flare-ups due to the application of the metal to the skin [35].

LTT is a method of testing a patient’s peripheral blood to detect memory T cells that react specifically to a certain metal to find metal-positive allergic reagents (Figure 3). The conventional LTT has low sensitivity and specificity, and is prone to false positives, making it inadequate for routine use compared to the patch test. However, LTT is an in vitro method using the patient’s blood, and if only a blood sample is taken, it is less physically demanding on the patient than the patch test, and there is no risk of new sensitization or allergic flare-ups due to metals. More research is required to improve and practicalize this approach by enhancing its sensitivity and specificity [36].

### 4.4. Diagnostic Techniques Used in Titanium Allergy Detection and Their Limitations

The patients in four and three articles were diagnosed using patch tests and LTT/MELISA, respectively. Patch tests and blood-based LTT/MELISA are currently used for the definitive clinical diagnosis of metal allergies [37,38]; however, both are known to yield false-positive and false-negative results in evaluations using titanium as the antigen, and are not completely reliable testing methods [9,11,14,34,39,40,41]. A systematic review examining the effectiveness of patch testing and LTT/MELISA in patients with suspected Ti hypersensitivity reported inconsistent results in terms of reliability and validity [42]. In addition, Ti allergy is historically new [43,44,45,46], and there are no uniform standards for the reagents and protocols used in the tests.

The use of Ti as an antigen for testing requires a solvent, and different solvents have been reported to produce different immunoreactions [34]. In a retrospective study, the commonly used agent TiO_2_ was reported to be likely to produce false-negative test results regardless of concentration, and titanium (IV) oxalate hydrate (TiC_4_O_9_H_2_-xH_2_O) was shown to drop to pH 2.0–3.0 when exposed to air and cause irritation, resulting in unstable test results [47]. In some studies, both TiCl_4_ and TiO_2_ were used; however, in Hosoki’s paper, the same patient did not react to TiO_2_ but showed positive results with TiCl_4_ in a patch test performed simultaneously [14]. Interestingly, none of the patients tested positive only for Ti in the patch test [15]. This may indicate a cross-reaction, which is commonly observed in allergic reactions [17]. It is also possible that the immune system is activated by the application of other metals, thereby making the patient more reactive, since patients who test positive for some metals may also be more sensitive to other metals [48]. In fact, patients who tested positive for Ti in patch tests also tested positive for highly antigenic metals, such as Ni and Hg (Table 2).

### 4.5. The Effect of Non-Titanium Metal Materials Included in Dental Implants

Titanium alloys (Ti-6Al-4V and Ti-6Al-7Nb) are used for implants because of their superior strength. However, because they are alloys, they also contain metals other than Ti. Even implants composed of pure titanium are known to contain trace amounts of various metals, such as aluminum, manganese, iron, beryllium, and nickel, which are not indicated [19,44,49,50,51]. Studies examining the in vivo release of trace elements from dental implant materials have identified low or very low levels of trace metals in various organs [49]. This may be related to the fact that patients who test positive for Ti also test positive for other metals. Therefore, more detailed examinations are essential to determine whether a suspected case of Ti allergy is really due to hypersensitivity to Ti itself; however, as mentioned above, no reliable and reproducible method has been established to replace the patch test and LTT/MELISA.

Some previous studies have also described good results with the removal of titanium implants and their replacement with zirconia implants [17,18]. Despite the absence of a definitive diagnostic method for titanium allergy, the alleviation of symptoms by the removal of the possible causative material indicates a cause-and-effect relationship. Müller-Heupt et al. suggested that the diagnosis of titanium hypersensitivity should not be based on a patch test or LTT/MELISA, but rather on the appearance of inflammatory clinical signs and the clinical course, which should be considered as the main parameters [42]. Nevertheless, a definitive diagnostic method is still essential for the clarification of this phenomenon.

Zirconia was identified as a valid alternative material in the selected articles and is now widely used in dentistry and for dental implants [52,53,54,55,56,57,58]. However, hypersensitivity reactions to zirconia, which was considered a safe biomaterial, have also been reported recently [59]. Since any material derived from a foreign source is essentially a foreign substance, the occurrence of foreign body reactions to these implants is not surprising. Thus, the clinical application of these materials should be performed with caution, especially in patients with a predisposition to allergies.

### 4.6. Other Factors That May Contribute to the Development of Titanium Allergy

The total number of male and female patients in the articles selected for this review was 90 and 280, respectively. Previous reports have indicated that metal allergy is more common in women, with a male-to-female ratio of 1:2–1:3 [60,61]. However, some reviews suggest that it depends on the exposure status in the past, and further studies on sex-related differences are required.

Malm et al. suggested that significant factors for early implant failure were systemic disease, allergies, smoking, analgesic medications other than nonsteroidal anti-inflammatory drugs (NSAIDs), implant-supported prosthesis in the opposite jaw, small bone volume, low primary stability, and healing complications [62]. The allergies included those to penicillin and other antibiotics, dust pollen, and plants as well as food allergies.

Penicillin allergy has also been identified as a risk factor in the articles summarized in Table 2 [20,21,22]. The case reported by Mitchel et al. also involved a history of a penicillin allergy [8]. Such cases of failure are considered to be associated with a high infection rate, and mainly involve early implant failures [1,63,64]. In the case reported by Mitchel et al., the patient was receiving steroids [8], which can further increase the risk of infection [65] and decrease bone density [66,67]. Since penicillin, other antibiotics, and steroids are risk factors for implant failure, such failures cannot be definitively attributed to titanium hypersensitivity.

Patients with some allergies are at risk of developing other allergies [68] and, therefore, may also be at risk of developing metal allergies. As mentioned, patients who test positive for any metal in a patch test, regardless of the type of implant placed, should be considered at risk of a hypersensitivity reaction.

## 5. Conclusions

Currently, there is no definitive method for diagnosing titanium allergy, which is a topic for future studies. Consequently, diagnoses of hypersensitivity to titanium are currently made on the basis of comprehensive data, including the clinical course and symptoms, results of clinical examinations using the patch test/LTT/MELISA, and confirmation of symptom relief by the removal of the implants. The findings of this study indicate the need to exercise caution while placing implants in patients with preoperative allergies.

## Figures and Tables

**Table 1:** Characteristics of the representative articles.

Year	Authors and References	Research method	Number of Patients	Clinical Symptoms	Diagnostic Tests	Clinical Outcomes
1990	Mitchell DL [8]	Case-report	2	Gingival hyperplasia	NA	Removed implants→ Symptom improvement
2006	Müller K [9]	Clinical and Experimental	56	Dermatitis, Eczema	MELISA/Patch test	Removed implants→ Symptom improvement
2007	du Preez LA [10]	Case-report	1	Bone resorption, Flank pain, Giant cell reaction	IHC	Removed implants→ Symptom improvement
2008	Sicilia A [11]	Case-control	1500	Dermatitis, Eczema, Pruritis	Patch test	Removed implants/Follow-up
2008	Egusa H [12]	Case-report	1	Eczema	LTT	Removed implants→ Symptom improvement
2011	Pigatto PD [13]	Case-report	1	Cheilitis	Patch test	Removed implants→ Symptom improvement
2016	Hosoki M [14]	Case-report	1	Eczema	Patch test (non Ti)	Removed implants→ Symptom improvement
2018	Hosoki M [15]	Clinical retrospective	16 implants	Eczema	Patch test	Removed implants and positive metals →Symptom improvement
2019	Anderei OC [16]	Case-report	1	Horizontal movement of an implant, Hyperplastic gingivitis	IHC	Removed implants→ Symptom improvement
2020	Tawil G [17]	Case-report	1	NA	LTT/MELISA	Removed Ti implants→ Replaced to Zr implants →Symptoms improvement
2021	Borgonovo AE [18]	Case-report	1	High mucosa sensitivity /Implant exposure	Biopsy/MELISA	Removed Ti implants→ Replaced to Zr implants →Symptoms improvement
2021	Alqahtani AR [19]	Case-report	1	Pain, Eczema, Swelling, Burning sensation	Medical history, Clinical symptoms after implantation	Removed implants/Follow-up

**Table 2:** Clinical treatments and course of symptoms.

Year	Authors	Number of Patients	Onset of Allergy Symptoms	Time of Removal	Time of Recovery, Course of Symptoms
1990	Mitchell DL [8]	2	Within 2 weeks after abutment placement 3.5 months after implant placement	16 months after abutment placement Not removed	Symptom resolution time unknown, no symptoms during 18 months of follow-up Symptom resolution time unknown, no symptoms during 11 months of follow-up
2007	du Preez LA [10]	1	1 week after implant placement	Not described	Not described
2008	Egusa H [12]	1	1 week after implant placement	2 years after implant placement	Symptoms disappeared after 10 months from removal
2011	Pigatto PD [13]	1	1 week after implant placement	Not removed	Not described
2016	Hosoki M [14]	1	2 years after implant placement, 6 months after placement of Ti screws in lower limb fracture	6 years after implant placement	Symptoms disappeared after 1 month from removal
2019	Anderei OC [16]	1	A few months	1.5 years after placement	Not described
2020	Tawil G [17]	1	A few days after implant placement	3 months after implant placement	Symptoms disappeared after 3 months from removal
2021	Borgonovo AE [18]	1	6 months after implant placement	Not described	Symptom resolution time unknown, no symptoms during 18 months of follow-up
2021	Alqahtani AR [19]	1	2 days after implant placement	Immediately	Symptoms disappeared after 3 weeks from removal

**Table 3:** Information on allergies other than Ti.

Positive Metals Other than Ti	Other Allergies	Sex	References
Ni, Hg		Female	[12]
Ni, Hg, Pd, Au, Cu		Female	[13]
Hg, Pd, Au, Cr, Co, Cu, Sn, Zn, Ir, Mo	Food	Male	[14]
Ni, Co		Female	[17]
NA	Pollen, Dust	Female	[18]
**Positive Metals Other than Ti**		**Patients or Implants**	**References**
Ni, Hg, Cd, Pd, Au, Pt, Sn		56 patients	[9]
Ni, Cr		1500 patients	[11]
Ni, Hg, Pd, Cr		16 implants	[15]

**Table 4:** Results of articles on penicillin allergy.

Year	Authors and References	Research Method	Patients or Implants	Suggestions
2016	French D [20]	Retrospective cohort study	5576 implants	Self-reported penicillin allergy is a risk factor for early implant failure due to high infection rates.
2018	Salomó-Coll O [21]	Cross-sectional study	1210 patients	Penicillin allergy is one of the risk factors for early implant failure.
2021	Block MS [22]	Retrospective case-controlled study	224 patients	Penicillin-allergic patients treated with other antibiotics showed four times the risk of suffering dental implant failure.

**Table 5:** Coombs and Gell classification of allergic reactions.

Classification	Immune Reactant	Timing of Reactions	Clinical Manifestations
Type I	IgE	Immediate Anaphylactic 15–20 min	Allergic rhinitis, Asthma, Food allergy Drug allergy, Anaphylactic shock
Type Ⅱ	IgG, IgM	Cytotoxic Various timings	Hemolytic transfusion reaction Autoimmune hemolytic anemia Immune thrombocytopenia
Type Ⅲ	IgG, IgM	Immune complex 3–12 h	Arthus reaction, Serum sickness SLE, Glomerulonephritis
Type Ⅳ	T cells	Delayed 24–72 h	Contact dermatitis Allograft rejection, Tuberculin reaction

## Data Availability

All relevant data are included within the paper itself.
